# GridCell: a stochastic particle-based biological system simulator

**DOI:** 10.1186/1752-0509-2-66

**Published:** 2008-07-23

**Authors:** Laurier Boulianne, Sevin Al Assaad, Michel Dumontier, Warren J Gross

**Affiliations:** 1 Department of Electrical and Computer Engineering, McGill University, Montreal, QC, H3A 2A7, Canada; 2 Department of Biology, Carleton University, Ottawa, ON, K1S 5B6, Canada

## Abstract

**Background:**

Realistic biochemical simulators aim to improve our understanding of many biological processes that would be otherwise very difficult to monitor in experimental studies. Increasingly accurate simulators may provide insights into the regulation of biological processes due to stochastic or spatial effects.

**Results:**

We have developed GridCell as a three-dimensional simulation environment for investigating the behaviour of biochemical networks under a variety of spatial influences including crowding, recruitment and localization. GridCell enables the tracking and characterization of individual particles, leading to insights on the behaviour of low copy number molecules participating in signaling networks. The simulation space is divided into a discrete 3D grid that provides ideal support for particle collisions without distance calculation and particle search. SBML support enables existing networks to be simulated and visualized. The user interface provides intuitive navigation that facilitates insights into species behaviour across spatial and temporal dimensions. We demonstrate the effect of crowing on a Michaelis-Menten system.

**Conclusion:**

GridCell is an effective stochastic particle simulator designed to track the progress of individual particles in a three-dimensional space in which spatial influences such as crowding, co-localization and recruitment may be investigated.

## Background

One of the main goals of computational cell biology aims to accurately simulate large biological systems at molecular resolution. Stochastic effects and spatial constraints are increasingly being recognized as important factors in the normal functioning of molecular networks [1]. The efficiency of biochemical networks is enhanced by component co-localization [2], and certain signaling networks are thought to be facilitated by transport and co-localization [3]. In addition, molecular crowding has been shown to affect biochemical systems [4-6]. Modeling and simulation of these kinds of networks requires new kinds of stochastic simulators.

**Figure 1:**
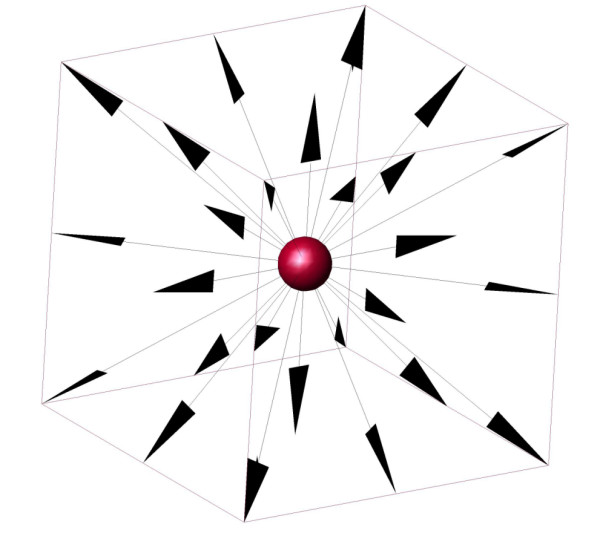
**D3Q27 cubic grid structure**. The 27 possible locations of a D3Q27 cubic grid that a given voxel can access.

We developed GridCell to simulate biological models with specific consideration for stochasticity, locality, and collision. GridCell is based on a simplified model for molecular movement and interaction. It uses a discrete three-dimensional cubic grid based on the D3Q27 model often used in the application of the Lattice-Boltzmann Method (LBM) [7]. Each voxel has access to itself and its 26 neighbours and is independent of voxels outside this immediate surrounding. Figure 1 shows the 27 possible locations accessible to a voxel from a D3Q27 grid. The integer-addressed 3D grid avoids floating-point computation and distance calculations, resulting in an efficient implementation. Molecules are represented as particles that move and react stochastically within discrete volumes in discrete timesteps. Collisions and molecular crowding are enforced since only one particle can occupy a given location at any time. GridCell stores the coordinates of all the particles on the 3D grid at every turn, thereby enabling particle tracking in both space and time.

The simulation space is visualized via a 3D interface and 2D graphs, and surface plots summarize molecule concentrations over space and time. GridCell supports models specified by the Systems Biology Markup Language (SBML) (please see Availability & requirements for further information). SBML models can be obtained from public repositories such as EBI's Biomodels database (please see Availability & requirements for further information) or designed using software such as SBMLeditor or JDesigner (please see Availability & requirements for further information).

## Implementation

### Algorithm

The simulation employs a two-phase process in which particles (1) attempt to move and then (2) attempt to react every turn.

#### Movement Phase

**Figure 2:**
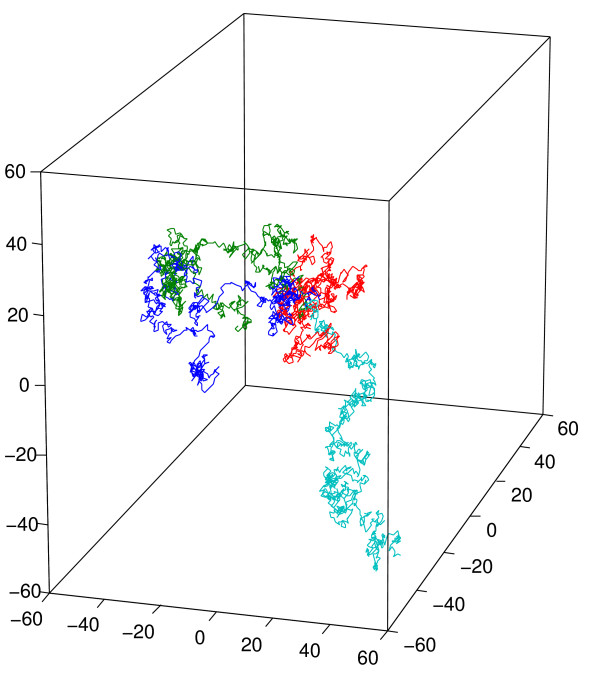
**Random Brownian walk**. Random walks of 4 different particles in GridCell after 1000 timesteps.

A particle can move at most once per timestep. Since a particle only has access to its immediate surrounding, a particle can only move in one of 27 nearest locations, including the current location. The selection of the movement direction is made randomly; therefore the particles follow a Brownian random walk. Figure 2 shows an example of 4 different Brownian random walks of particles starting from the same location. In GridCell, any particle attempting to move to an occupied location will generate a collision. A collision prevents the particle attempting to move from moving during that turn and does not affect the other particle.

#### Diffusion

Particles following a Brownian random walk should also follow the well-known Einstein-Smoluchowski equation

**Figure 3:**
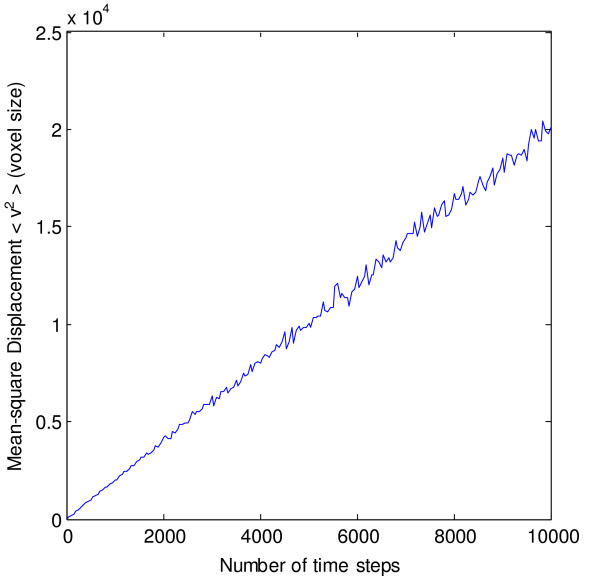
**Diffusion in GridCell**. Mean-square distance (over 1000 iterations) of particles versus the number of timesteps in GridCell.

(1) <*r*^2^> = 2*dDt*,

where <*r*^2^> is the mean-square displacement, *d *is the dimensionality, *D *the diffusion coefficient and *t *the elapsed time. Figure 3 shows the mean-square displacement in units of voxel <*v*^2^> (averaged over 1000 iterations) versus the number of timesteps *n*_*ts *_when the probability of movement of the particles at every timestep is equal to 1. As expected for an uncrowded case, the mean-square displacement increases linearly with the number of timesteps. This leads to the following relation

(2) <*v*^2^> = *An*_*ts*_,

where *A *is the slope of the graph. By substituting $<v^{2}>=\frac{<r^{2}>}{s_{vox}^{2}}$ and $n_{ts}=\frac{t}{l_{ts}}$ where *s*_*vox *_is the length of the sides of the voxels in meters and *l*_*ts *_is the length of the timestep in seconds, we get

(3) $$<r^{2}>=\frac{As_{vox}^{2}}{l_{ts}}t=2dDt.$$

Since the probability of movement at each timestep of the particle is equal to 1, *D *can be substituted for the maximum diffusion speed *D*_*max *_that GridCell can support for a given timestep and voxel size. This upper limit on diffusion speed is caused by the design decision of restraining particle movement to its immediate neighbourhood (the D3Q27 grid). By calculating the slope of the graph and setting the dimensionality *d *equal to 3, *D*_*max *_can be calculated as

(4) *D*_*max *_= 0.335*s*_*vox*_^2^/*t*_*s*_.

Smaller diffusion speeds are simulated by applying a different probability of movement such that

(5) *D *= *p*_*m*_*D*_*max*_,

where *p*_*m *_is the probability of movement of a particle at every timestep. As long as the diffusion speeds of the particles are smaller than *D*_*max*_, diffusion will be modeled correctly. If a larger diffusion speed is needed, one can reduce the timestep or increase the size of the voxels.

#### Reaction Phase

A particle may react only once per turn and only with its immediate surrounding. The reaction phase is completely independent from the movement phase, therefore it does not matter if a particle previously moved or collided with another particle. Common interactions include aggregation events such as molecular complex formation/dissolution or conversion events such as chemical reactions. Only the simplest reactions involving 3 or less particles are directly supported. Complex reactions involving more than 3 particles are decomposed into several elementary reactions. The probability of reaction per timestep is derived from the overall rate of reaction and is very similar to the approach taken by ChemCell [8]. Only 3 different reactions involve 3 or less participants: 1 reactant and 1 product, 1 reactant and 2 products and 2 reactants and 1 product. Let's consider the two reactions that involve a single reactant

(6) *A *→ *B*

(7) *A *→ *B *+ *C*.

Both reactions have a forward rate of reaction *k *in units of time^-1 ^and timestep is *t *second. Assuming a well-mixed approximation and *N *particles of type *A *in the system, then in both cases the expected number of reaction per turn is given by *N*(1 - *e*^-*kt*^). Considering each particle individually, each particle has a probability equal to 1 - *e*^-*kt *^to react during each timestep. In our stochastic model, a uniform random number *R*_*n *_between 0 and 1 is generated for each particle, and the reaction occurs if *R*_*n *_< 1 - *e*^-*kt*^. In a reaction with only 1 reactant and 1 product, the reactant is simply replaced by the product. In a reaction with 1 reactant and 2 products, a search is first conducted in the surrounding area. If there is at least 1 free voxel in the surrounding area of the particle, the reaction takes place, and the second product is positioned in that free location while the first product is placed at the position of the initial reactant. The reaction is blocked if no free position is found. This limitation only modifies the overall reaction rate of the reaction in a situation where the whole cell is completely filled which would prevent any movement and reaction to take place.

Consider the following reaction with 2 reactants:

(8) *A *+ *B *→ *C*,

with a rate constant *k *in units of (molarity*time)^-1 ^and a timestep between each iteration of *t *second. Assuming *N*_*a *_particle of type *A*, *N*_*b *_particle of type *B*, a Volume *V *and the Avogadro's number being *A*_*v*_, then the total number of reactions *N*_*r *_in a well-mixed system is given by

(9) $$N_{r}=\frac{kN_{a}N_{b}t}{A_{v}V}.$$

On average, the desired number of reactions in our system should be equivalent to the result of the above equation. In our system, particles can only react with their immediate surrounding locations. In a well-mixed system, the number of *A*,*B *pairs that are close enough to each other to generate a reaction is given by *N *= *N*_*a*_*N*_*b*_*V*_*c*_/*V *where *V*_*c *_is the volume of the cube containing the 26 "neighbouring" voxels and *V *is still the total volume of the simulation. If each of those pairs react with probability *P*, then *N*_*r *_= *NP*. Setting the 2 equations *N*_*r *_= *NP *= *kN*_*a*_*N*_*b*_*t*/(*A*_*v*_*V*) gives the equation

(10) $$P=\frac{kt}{A_{v}V_{c}}.$$

The formula is independent of *V*, *N*_*a *_and *N*_*b *_as expected. Also, for a given rate constant *k*, it is possible to have a set of parameter *t*/*V*_*c *_such that *P *is greater than 1. If that is the case, a smaller timestep or larger voxel size (proportional to *V*_*c*_) has to be selected. A random number *R*_*n *_is generated. If *R*_*n *_<*P*, then the first reactant will search its surrounding area for the second reactant. If it is found, the reaction takes place and the product is placed at the location of the first reactant. If no reactant is found, the reaction is aborted. Note that the second reactant does not try to react with the first one, doing so still enables us to get the right rate of reaction and reduces the number of operations needed to update the simulation. In the case where a particle can participate in many different reactions, a random number is generated to select which reaction is going to be tested first. If the first reaction does not take place, then the next reaction on the list is tested. The procedure will go on until either a reaction takes place or all possible reactions have been tried. This ensures that, on average, all reactions are tested equally.

When 2 reactants of the same species form a product such as

(11) *A *+ *A *→ *B*,

the individual rate of reaction of particle A needs to be modified to ensure that the overall rate of reaction is respected since each reactant will attempt to react with the other one. Assuming *y *is the overall probability of reaction and *x *is the individual probability of reaction of species A, then

(12) $$x=1-\sqrt{1-y}.$$

More complex reactions are implemented by creating a cascade of several elementary equations. This process, done automatically by the software, will break the complex reactions into a series of simpler reactions by introducing "temporary" species. For example, consider the following reaction with 1 reactant and 5 products.

(13) *A *→ *B *+ *C *+ *D *+ *E *+ *F*

where *k *is the rate of reaction in time^-1^. For each product exceeding 2, a temporary species is created. In this case, 3 temporary species are created. It follows that the reaction is broken down into:

(14) *T*_1 _→ *B *+ *C*

(15) *T*_2 _→ *D *+ *E*

(16) *T*_3 _→ *F *+ *T*_1_

(17) *A *→ *T*_2 _+ *T*_3_

where *T*_1_, *T*_2 _and *T*_3 _are respectively the first, second and third temporary species. By setting the rate of reaction of equation 17 equal to *k *and the probability of reaction of equations containing any temporary species on the reactant side equal to 1, we reduce the artifacts due to the creation of the "temporary" species to a minimum. Indeed, the temporary species disappear from the system as quickly as possible and the overall rate of reaction is identical.

Shown below is the case where more than 2 reactants merge into a single product:

(18) *A *+ *B *+ *C *+ *D *+ *E *→ *F*.

The procedure is similar to the previous case, 1 temporary species is created for each reactant above 2.

(19) *A *+ *B *→ *T*_1_,

(20) *C *+ *D *→ *T*_2_,

(21) *E *+ *T*_1 _→ *T*_3_,

(22) *T*_2 _+ *T*_3 _→ *F*.

where *T*_1_, *T*_2 _and *T*_3 _are respectively the first, second and third temporary species. In order to obtain the same overall probability of reaction and to reduce the impact of the temporary species on the system to a minimum, the probability of reaction of any reaction containing temporary species on the reactant side (equation 21 and 22) is set to 1. Assuming that *P *is the probability of reaction of the reaction presented in equation 18 and *P*_1 _and *P*_2 _are the probability of the first and second simple reactions *A *+ *B *→ *T*_1 _and *C *+ *D *→ *T*_2 _then, we set *P *= *P*_1_*P*_2_. We also set *P*_1 _= *P*_2_. Equating the 2 equations gives *P*_1 _= *P*_2 _= $\sqrt{P}$.

In general, the probability of the simple reactions *P*_*n *_containing no temporary species is equal to

(23) $$P_{n}=P\left\lfloor \frac{2}{N_{reactants}}\right\rfloor$$

where *P *is the probability of reaction and *N*_*reactants *_is the number of reactants of the initial reaction. Each temporary particle has a parameter *lifetime *which indicates the number of turns the particle has to live in the system before reverting back to its previous state. The short lifetime of temporary particles is important for 2 reasons. First, it makes sure that temporary particles are effectively temporary and never stay in the system for a long period of time. It also makes sure that all the reactants are to be close to each other in order for the reaction to complete. Usually, a lifetime of 2–3 turns is reasonable since it gives enough time to react with the neighbouring particles while making sure temporary particles do not constitute the bulk of the system.

Reversible reactions are handled by creating 2 different separate reactions, 1 for the forward reaction with the forward reaction rate and 1 for the backward reaction with the corresponding backward reaction rate. Assuming the following reaction

(24) *A *+ *B *+ *C *+ *D *+ *E *↔ *F*.

with forward reaction rate *k*_*f *_and backward reaction rate *k*_*b*_. This reversible reaction is then split into

(25) *A *+ *B *+ *C *+ *D *+ *E *→ *F*

with a reaction rate *k*_*f *_and

(26) *F *→ *A *+ *B *+ *C *+ *D *+ *E*

with reaction rate *k*_*b*_.

Temporary particles involved in a reversible reaction have a flag mentioning if they are participating in a forward or backward reaction such that they can revert back to the proper reactants when their lifetime reaches zero.

### Performance analysis

Preliminary tests have been conducted to determine how the software reacts to different system sizes. The tests have been executed on a stand-alone microprocessor: a 3.2 GHz P4 with 2GB of RAM. The current algorithm is computed serially. As it can be shown in Table 1, the time required to compute a timestep increases linearly with the number of particles and voxels present in the system. Tables 2 and 3 demonstrate how the performance is affected by independently modifying the number of voxels or the number of particles. The maximum number of particles that can be currently simulated is equal to the maximum number of voxels that can be supported, which is 10^7^. Table 4 shows that the number of reactions occurring at each timestep has a negligible effect on the performance. The reason is that all reactions have to be tested, regardless of whether or not they actually react. There are no practical limitations to the number of chemical species or the number of different reactions present in the system beyond the absolute limit on the number of voxels.

**Table 1:** GridCell performance versus system size

Number of Voxels	1e3	1e4	1e5	1e6
Number of Particles	3e2	3e3	3e4	3e5
Time (s)	1.62e-4	1.58e-3	1.6e-2	1.7e-1

Time required to compute a timestep versus the size of a simulation.

**Table 2:** GridCell performance versus number of voxels

Number of Voxels	1e3	1e4	1e5	1e6
Time (s)	1.6e-5	1.6e-4	2.14e-3	2.06e-2

Time required to compute a timestep versus the number of voxels. No particles in the system

**Table 3:** GridCell performance versus number of particles

Number of Particles	1e3	1e4	1e5	5e5
Time (s)	21.3e-2	26.4e-2	68.1e-2	22.8e-1

Time required to compute a timestep versus the number of particles. The system contains 1e6 voxels.

**Table 4:** GridCell performance versus the average number of reactions

Average Number of Reactions	0	16.5e3	29.5e3	39.5e3	47.5e3
Time (s)	7.4e-2	7.3e-2	7.25e-2	7.2e-2	7.1e-2

Time required to compute a timestep versus the average number of reactions occuring at each timestep. The system contains 1e6 voxels and 1e5 particles.

### User Interface Features

**Figure 4:**
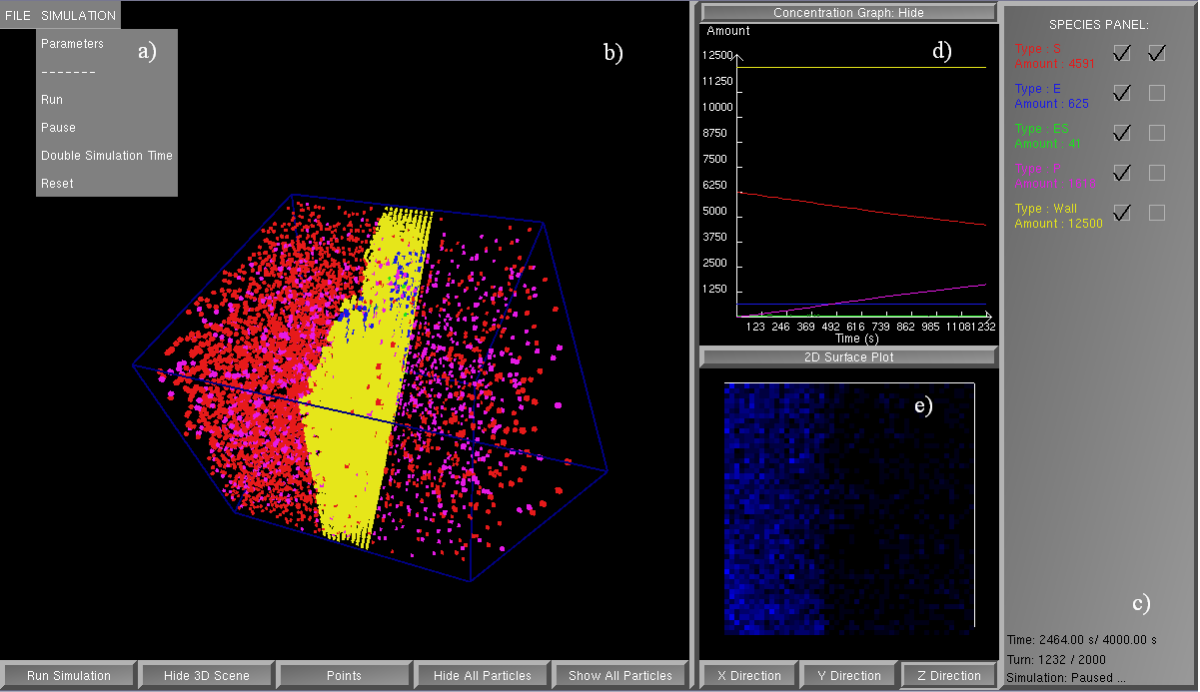
**GridCell user interface**. GridCell user interface with (a) menu, (b) 3D space, (c) species panel, (d) 2D plot of concentration versus time, and (e) 2D surface plot of concentration versus space. Simulated model involves the translocation of particles through a membrane with embedded enzymes.

The rendering is implemented in OpenGL, and most user-interface functions are written using the PLIB library, which is available online . GridCell's user interface (Figure 4) consists of a) a menu system, b) an interactive 3D simulation space, c) a species panel, d) a 2D plot of concentration versus time, and e) a 2D plot of concentration versus space.

The menu system (Figure 4a) provides the ability to load SBML models, set parameters and control the simulation. User-designated simulation parameters include the number of times to run the simulation, the timestep, the total simulation time, the sampling rate which is the frequency that the 2D graphs are updated and the results saved to file, and the frame rate which designates the frequency of updating the 3D visualization. GridCell computes the means and the standard deviations of the concentration over time if the user chooses to run multiple iterations of the simulation. These preferences may be saved and used later in any simulation. GridCell saves the particle concentrations and the 2D surface plot data in user-specified tab-delimited files. Spatial information such as specific compartment geometries or co-localization of particles is specified in an optional configuration file.

A key feature of the GridCell user interface is the ability to interact with the three-dimensional simulation volume (Figure 4b). Users can navigate into the 3D scene with mouse and keyboard controls to rotate, pan and zoom. Buttons are present to i) start/pause simulations, ii) change the particle representation from cubes to points for faster rendering, iii) turn off the visualization for optimal simulation performance, and iv) hide or show all particle types.

The species panel (Figure 4c) contains the current amount of each species, and allows species selection for the visualization plots. A second column specifies which species to render in the 2D surface plot of concentration versus space (Figure 4e). Particle colours are automatically selected from a predefined colour palette.

Finally, two plots to summarize particle concentrations with respect to time (Figure 4d) and space (Figure 4e) are provided in real-time. The 2D spatial plot displays increasing concentration with increasing brightness along a selected Cartesian axis.

## Results and discussion

### Michaelis-Menten reaction

The Michaelis-Menten equations are used to describe most enzymatic reactions. Its kinetics is given by the following equation:

(27) *E *+ *S *↔ *ES *→ *E *+ *P*.

The enzyme *E *reacts with the substrate *S *to form the complex *ES *with a rate of reaction *k*_1_. *ES *decomposes into the enzyme *E *and a new product *P *with a rate *k*_2_, or reverts back to its original form *E *+ *S *with rate *k*_*r*_.

#### Crowding

**Figure 5:**
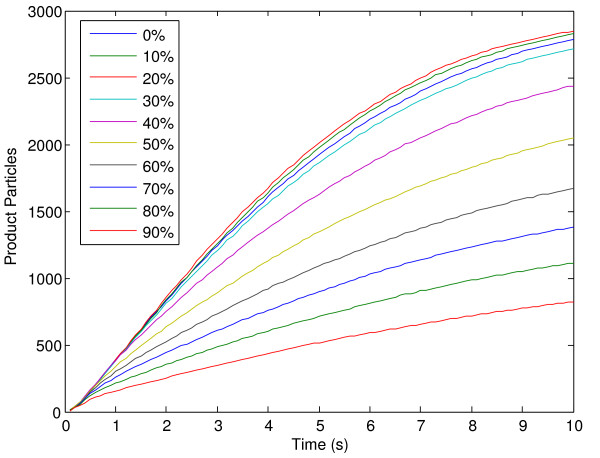
**Effect of crowding on Michaelis-Menten product formation using GridCell**. Effect of increasing the number of inert particles on product formation of a Michaelis-Menten system using GridCell. The mean has been calculated over 20 iterations. Percentage of voxels occupied by inert particles.

One of the main differences between GridCell and other simulators is its ability to simulate particle crowding. Molecular crowding occurs when particle density affects movement and reactivity. Crowding is typically ignored in most models since kinetics are often based on controlled, in vitro conditions that are not crowded. In addition, simulators do not typically support this feature since it is computationally expensive to keep track of all particle positions and their excluded volume, and to implement collision-detection algorithms. Some simulators (e.g. Smoldyn [9]) have shown crowding effects by explicitly introducing cubic obstacles [10] in the model. In contrast, GridCell implicitly exhibits molecular crowding effects by allowing inter-particle collisions. We demonstrate the effect of crowding by adding inert particles to a Michaelis-Menten system. Inert particles do not react with other molecules but reduce their movement and affect the overall number of reactions. The simulation parameters are described in Table 5. Figure 5 shows the number of products over time for a wide range of concentrations of inert particles averaged over 20 iterations. The individual simulations provided almost identical results to one another with a relative standard deviation smaller than 3.5% at the end of the simulation. The number indicated in the legend signifies the percentage of the voxels occupied by inert particles. In this specific example, with a voxel size of 3.2^-20 ^litres, this amounts to approximately 30000 inert particles per step of 10%.

**Table 5:** Simulation parameters

Volume (litres)	10^-14^
Number of *S *particles	3000
Number of *E *particles	1000
*k*_1 _(*M*^-1^*s*^-1^)	10^7^
*k*_2 _(*s*^-1^)	1
*K*_*r *_(*s*^-1^)	1
Simulation time (s)	10
Timestep (s)	10^-3^

Parameters of the Michaeles-Menten simulation.

Interestingly, the maximum rate of reaction is obtained when the inert particles occupy 20% of the volume, which agrees with the fact that macromolecular crowding may enhance reaction rates, as the particles have to search a smaller volume to find each other [11]. However, above 30%, the reaction rates decrease linearly as more and more inert particles are added. Under well-mixed and un-crowded systems, GridCell provides similar results to other ODE simulators and stochastic algorithms such as the stochastic simulation algorithm (SSA) from Gillespie [12] with the exception of a small stochastic noise [13].

### Related Work

GridCell is related to a family of Monte Carlo (MC) simulators (Table 6). SmartCell [14] and MesoRD [15] subdivide the simulation space into smaller subvolumes (voxels) that can contain many particles. Each subvolume is composed of a well-mixed solution, and particles can diffuse to adjacent subvolumes. This approach permits quicker simulations, but it is impossible to track individual particles, and molecular crowding has no effect on movement and reaction rates. Cell++ [16] combines a cellular automata engine with Brownian dynamics in order to simulate large quantities of small molecules on a discretized grid, while large molecules exhibit stochastic behaviour and move in a continuous space. Both spaces are then superimposed onto each other, and reactions can take place between the two different spaces. Currently, only collisions between particles (both small and large) and a fixed membrane separating two compartments are supported. Therefore, molecular crowding effects are not simulated. Unlike Cell++, MCell [17] tracks all individual particles in a continuous 3D space, and the diffusing particles follow Brownian dynamics. Particles may collide and interact with effector sites and 2D membrane surfaces, but not with other particles. ChemCell [8] calculates the probability of reaction at every timestep, and particles follow Brownian motion in a continuous space that requires a computationally expensive search algorithm to find nearby particles, and the use of dimensionless particles removes the ability of particles to collide with one another. In contrast, GridCell enables stochasticity-based investigations of SBML networks while considering spatial effects of recruitment, localization and crowding.

**Table 6:** Spatial simulators

	GridCell	SmartCell	MesoRD	Cell++	MCell	Smoldyn	ChemCell
Molecule representation	Particle	Population	Population	Large particles and populations of small particles	Particle	Particle	Particle
Stochastic	Yes	Yes	Yes	Large particles only	Yes	Yes	Yes
Space	Discretized	Discretized	Discretized	Continuous (large particles) and discretized (small particles)	Continuous	Continuous	Continuous
Particle-collision support	Yes	No	No	No	No	No	No
Diffusion support	Yes	Yes	Yes	Yes	Yes	Yes	Yes
SBML support	Yes	Yes	Yes	No	No	No	No
Web availability	Yes	Yes	Yes	Yes	Yes	Yes	No

Comparison and description of various simulators.

### Future Directions

GridCell performance is tightly linked to the number of voxels in the simulation space. The simulator can currently support a maximum of 10^7 ^voxels/particles which is not enough to simulate at a molecular resolution structures as complex as a complete cell, the long term goal of GridCell. However, the simple and regular algorithm of GridCell, which does not require any searches or complex operations, is a prime candidate for acceleration by parallelization to achieve performance speedup and simulate large-scale systems.

## Conclusion

GridCell is a stochastic simulator that uses a 3D grid and accounts for locality, very low concentration stochastic effects and particle collisions. Its user-interface makes it easy to use while providing several tools to analyze the system. GridCell reproduces the results obtained with ODEs and the Stochastic Simulation Algorithm (SSA) for simple systems when crowding and locality do not affect the system. We also show that particle collisions can have a significant impact on the speed of reaction and that the well-mixed assumption and dimensionless particles can induce a significantly different response in a biological system. The discrete 3D grid and the nearest-neighbour interactions remove the need to do any distance calculation, particle search and floating-point arithmetic. The regularity and simplicity of the algorithm makes it a good candidate for acceleration with a parallel architecture which will open the door to the simulation of even more complex systems.

## Availability and Requirements

The software is available at  and runs under the Windows XP operating system. This package includes sample SBML and GridCell configuration files. GridCell requires the Systems Biology Markup Language Library (libSBML 2.3.4-Xerces; ) and the OpenGL utility toolkit (GLUT 3.7.6; ). EBI's Biomodels database: . SBMLeditor: . JDesigner: . Systems Biology Markup Language (SBML): . 

## Authors' contributions

LB designed and programmed the GridCell simulator. SAA contributed to the programming of the GridCell simulator and its graphical user interface. MD and WJG contributed to the conception and design of the GridCell simulator. LB and SAA drafted the manuscript and MD and WJG revised the manuscript. All authors read and approved the final manuscript.
